# Surface-Based Regional Homogeneity in First-Episode, Drug-Naïve Major Depression: A Resting-State fMRI Study

**DOI:** 10.1155/2014/374828

**Published:** 2014-02-25

**Authors:** Hui-Jie Li, Xiao-Hua Cao, Xing-Ting Zhu, Ai-Xia Zhang, Xiao-Hui Hou, Yong Xu, Xi-Nian Zuo, Ke-Rang Zhang

**Affiliations:** ^1^Laboratory for Functional Connectome and Development, Magnetic Resonance Imaging Research Center, Key Laboratory of Behavioral Science, Institute of Psychology, Chinese Academy of Sciences, No. 16 Lincui Road, Chaoyang District, Beijing 100101, China; ^2^Department of Psychiatry, First Hospital of Shanxi Medical University, No. 85 Jiefang South Road, Taiyuan 030001, China; ^3^University of Chinese Academy of Sciences, Beijing 100049, China

## Abstract

*Background.* Previous volume-based regional homogeneity (ReHo) studies neglected the intersubject variability in cortical folding patterns. Recently, surface-based ReHo was developed to reduce the intersubject variability and to increase statistical power. The present study used this novel surface-based ReHo approach to explore the brain functional activity differences between first-episode, drug-naïve MDD patients and healthy controls. *Methods.* Thirty-three first-episode, drug-naïve MDD patients and 32 healthy controls participated in structural and resting-state fMRI scans. MDD patients were rated with a 17-item Hamilton Rating Scale for Depression prior to the scan. *Results.* In comparison with the healthy controls, MDD patients showed reduced surface-based ReHo in the left insula. There was no increase in surface-based ReHo in MDD patients. The surface-based ReHo value in the left insula was not significantly correlated with the clinical information or the depressive scores in the MDD group. *Conclusions.* The decreased surface-based ReHo in the left insula in MDD may lead to the abnormal top-down cortical-limbic regulation of emotional and cognitive information. The surface-based ReHo may be a useful index to explore the pathophysiological mechanism of MDD.

## 1. Introduction

Major depressive disorder (MDD) is characterized by cognitive deficits, functional disability, and pervasive negative feelings, such as sadness, anxiety, dysphoria, anhedonia, sleep abnormalities, and feelings of worthlessness [1]. MDD patients have deficits in emotional self-regulation and are prone to negative emotion bias toward life events [2]. Researchers have used behavioral, neurochemical, and electrophysiological approaches to explore the pathophysiology of depression, but the mechanisms of pathophysiology are still unclear.

Most recently, resting-state fMRI (RFMRI) has been widely used to study the functional brain abnormalities of MDD, given the highly synchronous nature of spontaneous low-frequency oscillations (0.01–0.08 Hz) in motor cortices [3]. Of the many RFMRI approaches, regional homogeneity (ReHo) has attracted substantial attention in research studies. ReHo employs Kendall's coefficient of concordance (KCC) to measure the functional coherence of a given voxel with its nearest voxels in a voxelwise analysis; it assumes that the spontaneous neural activity of a given voxel is similar to its neighbors [4–6]. ReHo reflects the temporal homogeneity of the regional blood oxygen level dependent (BOLD) signal. Thus, an abnormal ReHo may be related to changes in temporal spontaneous neural activity of a certain region [4].

Various studies explored spontaneous brain activity in MDD with the volume-based ReHo approach. The MDD group included medicated and unmedicated patients and showed decreased ReHo mainly in the frontal and limbic lobes and the basal ganglia [7]. Given that the MDD group included some medicated patients, the results could not exclude a potential medication effect. Two other studies explored the ReHo in treatment-resistant depression (TRD) patients and found that TRD patients showed lower ReHo in the left insula, the superior temporal gyrus, the inferior frontal gyrus, the posterior fusiform gyrus, the superior parietal lobule, and the precuneus [8, 9]. In these two studies, all of the patients were medicated and treatment-resistant; thus, the medication may have contributed to the results. Our previous study investigated the ReHo in 15 first-episode, drug-naïve MDD patients, 15 first-degree relatives, and 15 healthy controls; the results revealed that both MDD patients and the first-degree relatives showed decreased ReHo in the right insula and the left cerebellum [10]. Two other studies investigated first-episode and drug-naïve MDD patients and reported decreased ReHo in the left thalamus, the left temporal lobe, the left cerebellar posterior lobe, and the bilateral occipital lobe [11] and increased ReHo in the fusiform gyrus and the bilateral superior frontal gyrus in comparison with healthy controls [12].

These previous ReHo studies on MDD are based on the 3-dimensional volume-based calculation. Although volume-based activation maps have been used widely to date, this approach neglected the intersubject variability in cortical folding patterns. Therefore, some anatomical regions could not accurately match the same location in the standard template. While under the cortical surface space, the anatomical regions could occupy the same place, reduce the intersubject variability, and increase statistical power [13–15]. Recently, a volume-based ReHo computation was transplanted into a 2-dimensional variant with cortical surface-based fMRI analysis; the surface-based ReHo was found to have moderate to high intersession and intrasession test-retest reliability [16].

In the present study, using this relatively new surface-based ReHo approach, we aimed to explore whether the surface-based ReHo could be an effective biomarker of MDD. We recruited first-episode and drug-naïve patients to exclude a potential medication effect. Moreover, we also aimed to investigate the relationships between the ReHo and the clinical variables in the MDD group.

## 2. Methods

### 2.1. Participants

Thirty-two patients with MDD from 17 to 60 years of age were recruited from the inpatient and outpatient units in the Department of Psychiatry, First Hospital of Shanxi Medical University. All of the patients were diagnosed with a Chinese version of the Modified Structured Clinical Interview for DSM-IV for patient version (SCID-I/P) [17] by two trained clinical psychiatrists. All MDD patients were drug-naïve and in their first episode of illness (the first time they experienced a depressive episode). These patients had no history of other major psychiatric illness, neurological illness, head injury, and alcohol or drug abuse. MDD patients were rated with a 17-item Hamilton Rating Scale for Depression (HRSD) before the imaging scan.

Thirty-three age-, gender-, and education-matched healthy controls were recruited by advertisements. They were interviewed with the Structured Clinical Interview for DSM-IV for nonpatient version (SCID-I/NP). All healthy controls were free of depression or other psychiatric or neurological illness and had no history of head injury and alcohol or drug abuse.

All MDD patients and healthy controls were right-handed. Written informed consent was obtained from each participant. The Medical Research Ethics Committee of the First Hospital of Shanxi Medical University approved this study.

### 2.2. Data Acquisition

Participants were scanned by the 3.0T Siemens Trio scanner. During the scanning, participants laid supine in the scanner with their heads fixed with foam pads to decrease head motion and reduce scanner noise. They were informed to close their eyes and remain awake while moving as little as possible. The scanning sessions included the following: (i) three-dimensional T1-weighted whole-brain images: 3D-MPRAGE sequence, TR/TE = 2300/2.95 ms, 160 slices, thickness/gap = 1.2/0.6 mm, FOV = 226 × 240 mm, matrix = 240 × 256, and flip angle = 9° and (ii) the resting-state fMRI image: echo planar imaging (EPI) pulse sequence, 32 slices, TR/TE = 2000/30 ms, thickness/gap = 3/1 mm, matrix = 64 × 64, FOV=240 × 240 mm, flip angle = 90°, and 212 volumes.

### 2.3. Data Preprocessing

The Connectome Computation System (CCS: http://lfcd.psych.ac.cn/ccs.html) carries all steps of preprocessing both the structural and functional image preprocessing [16]. The structural image processing included the following steps: (1) the MR image denoise with a spatially adaptive nonlocal means filter [18, 19], (2) reconstruction of cortical surface, (3) segmentation of the cerebrospinal fluid (CSF), white matter (WM), and gray matter (GM) volumetric structures, (4) estimation of a triangular mesh tessellation over the GM-WM boundary and the mesh deformation to produce a smooth representation of the GM-WM interface (white surface) and the GM-CSF interface (pial surface) spatial normalization from individual native space to *fsaverage* stereotaxic space, (5) correction of topological defect on the surface, (6) inflation of individual surface mesh into a sphere, and (7) estimation of the deformation between the resulting spherical mesh and a common spherical coordinate system. The functional image preprocessing included (1) drop of the first five volumes, (2) slice timing correction, (3) 3D motion correction, (4) 4D global mean-based intensity normalization (mean intensity: 10,000), (5) nuisance regression (the WM and CSF mean time series and the Friston-24 motion time series) [20], (6) band-pass filtering (0.01–0.1 Hz), (7) removal of linear and quadratic trends, (8) coregistration between individual structural and functional images by the GM-WM boundary-based registration (BBR) algorithm [21], and (9) projection of the individual preprocessed 4D RFMRI time series onto a standard cortical surface *fsaverage5*.

Following the preprocessing, a data quality control procedure (QCP) was conducted. The QCP of structural images included visual head motion inspection, tissue segmentation, and pial and white surface reconstruction. The QCP of functional images included steps of checking of the minimal cost of coregistration (mcBBR) and the root mean square of framewise displacement (rmsFD) (http://lfcd.psych.ac.cn/ccs/QC.html).

### 2.4. Surface-Based ReHo Analysis

Surface-based ReHo analysis was performed with the CCS. Details of the analysis can be found in our recent study [16]. The individual preprocessed R-FMRI data was projected into the *fsaverage5* (FREESURFER 5.1) surface space. For each vertex of the surface space, the corresponding coordinates were computed in anatomical and functional space, and then the trilinear interpolation was used to interpolate the fMRI values [16]. Surface-based ReHo was calculated by Kendall's coefficient of concordance of the time series of a given vertex with those of its nearest neighbors. This computational procedure was repeated for all vertices in surfaces of both hemispheres to produce vertexwise KCC-ReHo surface maps, which are denoted 2-dimensional ReHo (2dReHo). When 7 nearest neighbors were used to explore the 2dReHo, we call it 2dReHo. Moreover, we also used a broader range of 19 neighbors to calculate 2dReHo and named it 2dReHo2. The individual 2dReHo maps were smoothed with a Gaussian kernel of 6 mm full-width half-maximum for subsequent statistical analyses.

### 2.5. Statistical Analysis

Unpaired two-sample *t*-tests were performed to compare age and years of education. A chi-square test was used to compare the gender ratio between the two groups. Voxelwise one-way ANCOVA tests (covariates: age, sex, years of education, mcBBR, and rmsFD) were performed to explore the 2dReHo differences between the MDD and healthy controls. The Pearson correlation coefficients between each vertex on the surface and demographic and clinical variables were calculated. We employed a cluster-level familywise error (FWE) correction for multiple comparisons [22]. Specifically, given a vertexwise statistical parameter map, a vertexwise *P* value (i.e., uncorrected *P* < 0.01) was first assigned to form clusters across the entire hemisphere. Based upon the distribution of spatial extents of the survived clusters, an implicit cluster size or extent was generated to achieve the final corrected *P* value (here, *P* < 0.05) for controlling the FWE.

## 3. Results

### 3.1. Participants

Participants' characteristics are shown in Table 1. The two groups were matched by age, gender ratio, and years of education. Moreover, age at illness onset, illness duration, and HRSD score of MDD patients are also included in Table 1.

### 3.2. Surface-Based ReHo Differences between MDD and Healthy Controls

**Figure 1:**
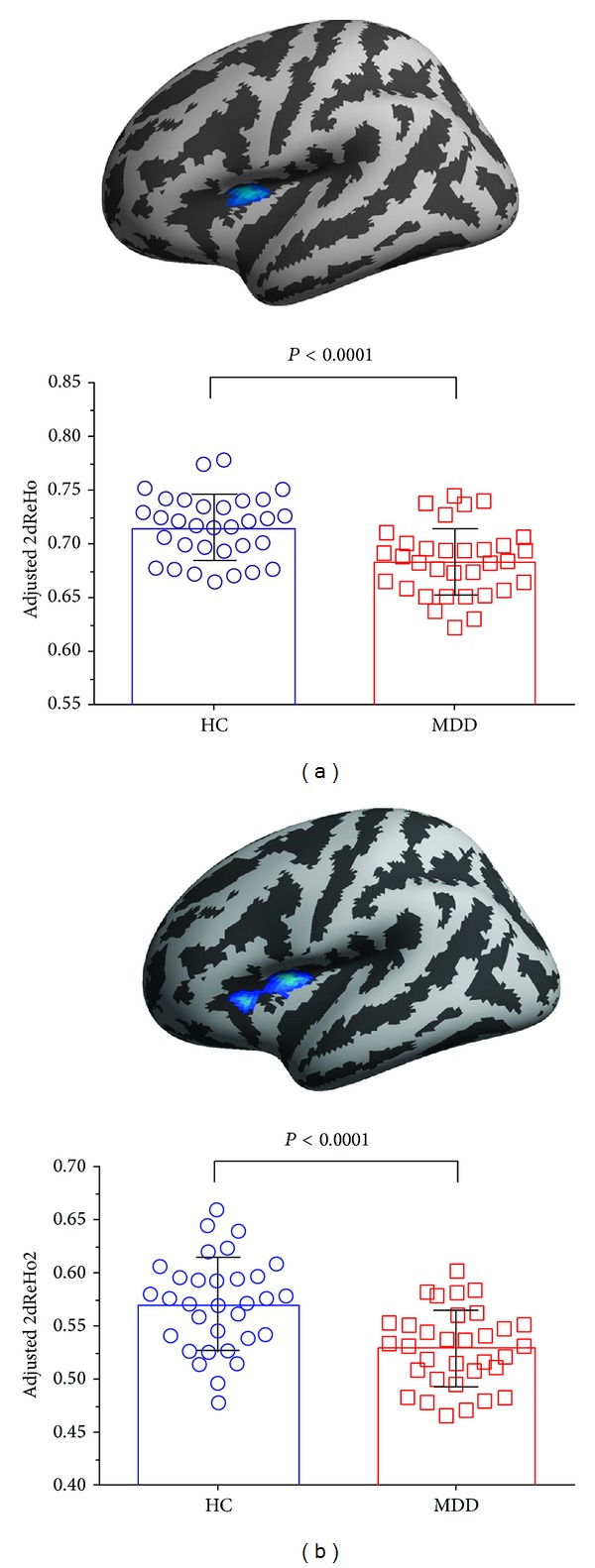
MDD patients showed lower vertexwise functional homogeneity in left insula ((a) 2dReHo and (b) 2dReHo2) compared to healthy controls.

Across the cortical surface, no significant differences were found between the MDD patients and healthy controls for the global mean ReHo or ReHo2 (Table 1). Moreover, MDD patients had a significant decrease in ReHo and ReHo2 in the left insula when compared with healthy controls (Figure 1). There was no increase in ReHo or ReHo2 in MDD patients.

### 3.3. Correlation Analysis

For MDD group, there were no significant correlations between age/years of education with left insula ReHo values (age: *P* = 0.26 and *P* = 0.26 for ReHo and ReHo2; years of education: *P* = 0.81 and *P* = 0.68 for ReHo and ReHo2, resp.). Moreover, no significant correlations were found between the left insula ReHo values and the patients' age at illness onset (*P* = 0.24 and *P* = 0.25), illness duration (*P* = 0.90 and *P* = 0.99), or the HRSD total score (*P* = 0.41 and *P* = 0.43).

We also calculated the correlations between global ReHo values and the age and years of education. For MDD group, no significant correlations were found between age/yeas of education and global ReHo values (age: *P* = 0.36 and *P* = 0.44 for ReHo and ReHo2; years of education: *P* = 0.43 and *P* = 0.40 for ReHo and ReHo2). For healthy control group, there were also no significant correlations between age/years of education and global ReHo values (age: *P* = 0.25, *P* = 0.29 for ReHo and ReHo2; years of education: *P* = 0.72, *P* = 0.72 for ReHo and ReHo2).

## 4. Discussion

To the best of our knowledge, this is the first study of surface-based ReHo in MDD. The present results reveal that MDD patients show decreased surface-based ReHo in the left insula; no significant correlations were found between the surface-based ReHo value in the left insula and the clinical characteristics of MDD.

The present surface-based ReHo results are more concise and partly supported by previous volume-based ReHo studies in MDD [8, 10]. Our previous volume-based ReHo study found that first-episode, drug-naïve MDD patients had decreased volume-based ReHo in the right insula cortex [10]. Different approaches may be the main reason for the differences across studies. Moreover, the participants and the scanning parameters were also different. The present results are supported by a recent meta-analysis of resting-state brain activity in MDD [23]. This meta-analysis included resting-state fMRI studies analyzed by volume-based ReHo and independent component analysis, as well as several PET studies of MDD; the results revealed that MDD patients had lower brain activity in left insula in comparison with healthy controls [23].

Located among the frontal, temporal, and parietal lobe and limbic regions, insula cortex is considered as an integration center of external events and internal cognitive processing [24, 25] and plays important role in various emotional and cognitive functions [26]. Compared with healthy controls, MDD patients had decreased activation in the left insula in a series of emotion-related tasks [27–29]. Recent RFMRI studies also reported decreased functional connectivity between the left insula and the bilateral amygdala [30] and the insula cortex and the subgenual anterior cingulate cortex [31] in MDD patients. Another RFMRI study further revealed that nonrefractory depression was associated with decreased functional connectivity in the limbic-striatal-pallidal-thalamic circuits, of which the insula is an important part [32]. A recent meta-analysis focusing on cortical-subcortical interactions in emotion demonstrated that the insula is activated across all basic and social emotions [33]. Two other reviews also highlighted the insula as an important region involved in emotional processing and the affective symptoms in MDD [34, 35]. Following a systematic review of the role of the insula cortex in MDD, researchers proposed that, due to the neuroanatomical connections, the insula might also be part of the frontolimbic network [36]; this network plays a very important role in emotion regulation. The decreased activity in the insula cortex of MDD may lead to somatic complaints, emotional dysfunctions, and the negative bias in explaining life events [31]. The abnormal activity of the corticolimbic network leads to the disruptions of top-down processing, which is thought to mediate pervasive emotions of sadness and negative affect in MDD patients.

Recent neuroanatomical and neurochemical studies reveal that the insula cortex plays an important role in the pathophysiology of MDD. MDD patients show significantly reduced volume [37] and cortical thickness [38] in the insula cortex. A voxel-based morphometry meta-analysis supports evidence that the clinical high-risk populations for psychosis show reduced gray matter in the left insula [39]. MDD patients also show lower fractional anisotropy in the left insula compared to healthy controls [40]. Several positron emission tomography (PET) studies found that MDD patients showed increased 5-HTT binding potential [41], decreased binding of the metabotropic glutamate receptor [42], and lower rCBF levels [43] in the insula cortex compared to healthy controls.

The correlation analysis shows that the surface-based ReHo value in the left insula cortex was not significantly correlated with the clinical variables and the depressive symptoms. Of the previous volume-based ReHo studies in MDD, only one study reported correlations between the ReHo value in the right insula and the severity of anxiety [7]; several other studies did not find significant correlations between the ReHo values and the clinical variables, as well as the HRSD score in first-episode and drug-naïve MDD [10], early-onset and late-onset drug-naïve MDD [12], TRD [8], and late-life depression [44]. The structural imaging studies also provided inconsistent results. No significant correlations were found between insula volume and the severity of depressive symptoms [37], while significant correlations were reported between the insula cortex and the Beck Depression Inventory, the Hamilton Depression Rating Scale, and the Snaith-Hamilton Pleasure Scale in MDD patients [45]. Together with these correlation results, the correlations between the ReHo values in the potential biomarker regions and the depressive symptoms still require further exploration.

Although the present study focused on the surface-based ReHo, we also analyzed the volume-based ReHo in the same cohort of participants. With the same covariates (age, sex, years of education, mcBBR, and rmsFD) and the same statistical criteria (corrected, *Z* = 2.3, *P* < 0.05), we found no significant clusters between MDD and healthy controls with volume-based ReHo approach. Some technical and methodological reasons may result in the differences between surface- and volume-based ReHo approaches. Firstly, the volume-based ReHo measures local signal synchronization in both gray and white matter, while the surface-based ReHo measures mostly the gray matter local synchronization signals. Therefore, the volume-based ReHo may include some artificial signals from white matter. Secondly, for 2dReHo and 3dReHo, although we try to keep the same length of neighbors for a certain vertices or voxel, the number of neighbors is different; the 2dReHo1 and 2dReHo2 calculation recruits 6 and 19 neighbors, respectively, while the 3dReHo recruits 26 neighbors. Moreover, the mask templates are different (MNI 152 3 mm standard volume for 3dReHo, while *fsaverage5* 4 mm resolution standard space for 2dReHo). These factors may influence the results in some degree. Thirdly, on the cortical surface, the intersubject variability in anatomy and the intersubject registration in both brain structure and function may be estimated more accurately [16]. Finally, previous volume-based neuroimaging studies consider that some subcortical regions (amygdala and thalamus) play important role in emotional circuit [46, 47]; however, the present surface-based ReHo only investigates the cortical regions; therefore it cannot reveal the differences in subcortical regions between healthy controls and MDD.

Although the present surface-based ReHo revealed interesting results, this study also had several limitations. First, previous findings suppose that MDD patients show deficits in the prefrontal-amygdala-pallidostriatal-mediothalamic mood regulating circuit [46, 47]. Given that the present surface-based ReHo approach focuses only on the cortical cortex, we did not examine the surface-based ReHo in the subcortical cortex, which are important regions of the mood regulating circuit. Second, the present study did not include neuropsychological tasks, specifically, the emotion processing related tasks. Including these tasks may help to determine whether the first-episode, drug-naïve MDD patients show deficits in the corresponding behavioral levels.

In conclusion, the present study revealed decreased surface-based ReHo in the left insula in MDD patients. The abnormal activity in the left insula may lead to the abnormal top-down cortical-limbic regulation of the emotional and cognitive based information. Moreover, no significant correlations were found between the surface-based ReHo value in the left insula and the age, years of education, age of illness onset, illness duration, and HRSD score in MDD patients.

## Figures and Tables

**Table 1:** Participants information.

	MDD (*N* = 33)	Health control (*N* = 32)	*P*
Age (years)	34.18 (10.96)	34.56 (9.92)	0.88^a^
Gender (male/female)	13/20	13/19	0.92^b^
Yeas of education	13.18 (3.09)	13.72 (2.93)	0.48^a^
Illness onset age	33.91 (10.92)		
Illness duration (month)	6.02 (6.25)		
HRSD	20.16 (3.22)		
mcBBR	0.59	0.60	0.82^a^
rmsFD (mm)^c^	0.14	0.14	0.55^a^
Global ReHo	0.67	0.66	0.51^a^
Global ReHo2	0.52	0.52	0.63^a^

MDD: major depressive disorder; HRSD: Hamilton Rating Scale for Depression. The values in brackets are standard deviations.

^a^Obtained by two-sample *t*-test. ^b^Obtained by chi-square test; ^c^rmsFD is the root mean square of the framewise displacement for in-scanner head motion.
